# A new strategy to minimize humidity influences on acoustic wave ultraviolet sensors using ZnO nanowires wrapped with hydrophobic silica nanoparticles

**DOI:** 10.1038/s41378-022-00455-2

**Published:** 2022-11-15

**Authors:** Yihao Guo, Jian Zhou, Zhangbin Ji, Yanghui Liu, Rongtao Cao, Fengling Zhuo, Kaitao Tan, Huigao Duan, Yongqing Fu

**Affiliations:** 1 College of Mechanical and Vehicle Engineering, Hunan University, Changsha, China; 2 Faculty of Engineering and Environment, Northumbria University, Newcastle upon Tyne, UK

**Keywords:** Electrical and electronic engineering, Nanowires

## Abstract

Surface acoustic wave (SAW) technology has been widely developed for ultraviolet (UV) detection due to its advantages of miniaturization, portability, potential to be integrated with microelectronics, and passive/wireless capabilities. To enhance UV sensitivity, nanowires (NWs), such as ZnO, are often applied to enhance SAW-based UV detection due to their highly porous and interconnected 3D network structures and good UV sensitivity. However, ZnO NWs are normally hydrophilic, and thus, changes in environmental parameters such as humidity will significantly influence the detection precision and sensitivity of SAW-based UV sensors. To solve this issue, in this work, we proposed a new strategy using ZnO NWs wrapped with hydrophobic silica nanoparticles as the effective sensing layer. Analysis of the distribution and chemical bonds of these hydrophobic silica nanoparticles showed that numerous C-F bonds (which are hydrophobic) were found on the surface of the sensitive layer, which effectively blocked the adsorption of water molecules onto the ZnO NWs. This new sensing layer design minimizes the influence of humidity on the ZnO NW-based UV sensor within the relative humidity range of 10–70%. The sensor showed a UV sensitivity of 9.53 ppm (mW/cm^2^)^−1^, with high linearity (*R*^*2*^ value of 0.99904), small hysteresis (<1.65%) and good repeatability. This work solves the long-term dilemma of ZnO NW-based sensors, which are often sensitive to humidity changes.

## Introduction

Surface acoustic wave (SAW)-based sensors detect changes in the resonant frequencies of acoustic waves using interdigital transducers (IDTs) made on the surface of a piezoelectric substrate^1,2^. As acoustic wave energy is confined to the surface of the device within a thickness range of 1–2 wavelengths, these SAW devices are very sensitive to changes in surface properties, e.g., mass loading^3^, viscosity^4^, conductivity^5^, temperature^6^, and humidity^7^ changes. Therefore, SAW sensors have received substantial attention to detect physical parameters including mass^8^, strain^9^, ultraviolet (UV)^10^, etc., or electrochemical and biological signals, such as concentrations of gas^11^, vapors^12^, ions^13^, DNA, and proteins^14,15^. They have the advantages of relatively high sensitivity, miniaturization, ready integration with microelectronics, convenient operation, fast response, excellent stability, and passive/wireless ability^16,17^.

UV radiation refers to electromagnetic waves with wavelengths of 100–400 nm that have profound influences on human health. For example, sunlight, which contains UV light, is healthy for the human body, as it promotes the synthesis of vitamin D, but excessive exposure to sunlight can lead to skin cancer. In addition, UV light with a wavelength of 365 nm can inactivate *E. coli* and mesophilic bacteria and has been used for water disinfection^18,19^. Therefore, the detection of UV light can monitor the germicidal efficiency of UV radiation.

Recently, SAW technology has been extensively investigated for UV detection based on the acoustoelectric effect^20^, mass-loading effect, and photo-capacitive effect^21^. Compared with other types of UV sensors, SAW sensors have many advantages, such as easy integration with integrated circuits (ICs), miniaturization, portability, a uniquely remote wireless operation capability, and potentially zero power consumption^22^.

For high-performance SAW UV detection (e.g., with great sensitivity or a fast response), a layer of sensing material such as ZnO has often been used because of its outstanding properties and ease of preparation. ZnO has a direct bandgap of 3.37 eV, which makes it highly suitable for UV detection. ZnO also has good radiation resistance because of its wide bandgap, thus making it highly suitable for use in harsh environments^21^. Commonly used ZnO structures include ZnO thin films^23^ and recently, ZnO nanostructures, such as nanorods^24^, nanowires^7^, nanosheets^25^, nanobelts^26^, nanoflowers^27^ and nanoparticles^28^. Compared with ZnO thin films, ZnO nanostructures offer unique advantages, including a larger specific surface area, higher excitation bonding energy, and stronger electrochemical activity, thus leading to better UV detection sensitivity^29^. Among these ZnO nanostructures, ZnO nanowires are commonly applied because nanowires with various morphologies can be easily produced^22^.

For UV sensing using ZnO nanowires, Peng et al. synthesized ZnO nanowires using a thermal evaporation method and then fabricated a SAW UV detector^22^. They reported an average central frequency shift of over 65 kHz under 150 μW/cm^2^ 365 nm UV light illuminated for several on–off cycles. Mohanan et al.^30^ proposed an on-chip technique for the direct growth of ZnO NWs on selected sites of a SAW resonator through a self-seeding thermal evaporation method, and the sensitivity of this ZnO NWs UV sensor with a 365 nm wavelength UV light source was 0.26 ppm (mW/cm^2^)^−1^. Yin et al. applied graphene quantum dot-decorated ZnO NW to enhance the UV sensing performance of a flexible and transparent SAW photodetector made on flexible glass. This flexible UV sensor showed a sensitivity of 1.66 ppm (mW/cm^2^)^−1^ and maintained good performance under a bending angle of ∼30° after 200 cycles without apparent degradation, showing excellent flexibility^10^.

However, ZnO is normally hydrophilic, and thus ZnO NWs easily form loose and porous interconnected 3D network structures due to their specific surface areas and porosities. In a humid environment, water molecules are easily absorbed onto these ZnO nanostructures, which causes significant mass loading on the device’s surface and large frequency shifts. Therefore, ZnO materials are often used as a humidity-sensitive layer for SAW humidity sensors. For example, Wu et al. prepared a highly flexible and ultrasensitive ZnO/glass SAW humidity sensor on ultrathin glass with ZnO NWs and a graphene quantum dot composite sensitive layer and reported humidity sensitivity of up to 40.16 KHz/%RH^7^. Tao et al. used three-dimensionally quadruped ZnO flowers as the sensitive layer to enhance the UV sensing performance of a SAW device and enhanced humidity sensitivity up to 2.9-fold^31^. As discussed above, when ZnO NWs are used as the sensitive layer for UV sensors, a change in humidity will significantly affect the detection sensitivity of the sensor for UV signals and cause large measurement errors (or noise). However, the effects of UV radiation and humidity on frequency shifts are often combined and cannot be easily separated. This causes a large issue for the real-time monitoring of UV signals using ZnO NWs as a sensing layer under various environmental humidity conditions. This challenge therefore motivates the development of new materials, new rational device architectures, and new manufacturing methods to solve this dilemma.

**Fig. 1:**
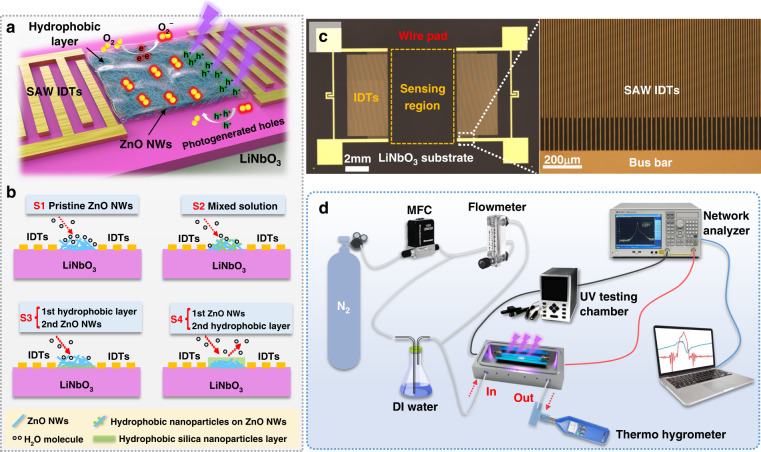
Schematic illustration of UV detection on the proposed SAW sensitive layer and sensing test system. **a** A schematic illustration of UV detection using a SAW device with the proposed novel sensitive layer. **b** Four different strategies to construct a ZnO NWs sensing layer for the SAW UV sensor. **c** An optical microscope image of the fabricated SAW device and IDTs. **d** System diagram for UV sensing in various humidity environments

In this paper, we proposed a novel strategy by applying hydrophobic silica nanoparticles wrapped on the surface of ZnO NWs. The design methodology for this sensing layer is shown in Fig. 1a, which effectively integrated the merits of these two types of nanomaterials; thus, the fabricated ZnO NW-based UV sensors show insignificant interference over a wide range of relative humidity (RH) values of 10∼70%. It has high UV sensitivity, superior repeatability, good linearity and small hysteresis.

## Experimental section

### Synthesis and characterization of the hydrophobic ZnO NWs sensing material

ZnO NWs powder (4 mg, purchased from Xianfeng Nanotechnology Co., Ltd., China) was first added to 20 ml of deionized (DI) water and then magnetically stirred for 2 h to obtain a ZnO NWs solution with a ZnO NWs concentration of 0.2 mg/ml.

To prepare hydrophobic silica nanoparticles, aqueous ammonia (NH_3_, 28%), anhydrous ethanol, tetraethyl orthosilicate (TEOS, 99.99%), and 1H,1H,2H,2H-perfluorooctyltriethoxysilane (POTS, 97%) were used, all of which were purchased from Aladdin Industrial Corporation, China. The preparation procedures are illustrated in Fig. S1^32–34^. First, a mixed solution (including 50 mL of anhydrous ethanol and 2.5 mL of ammonia water) was placed in a water bath at 30 °C, and then 1.25 mL of TEOS was added. After continuous stirring at 200 r/min for 10 h, a suspension of silica nanoparticles was obtained. Additionally, 45 mL of anhydrous ethanol and 1 mL of POTS were mixed in a 150 mL beaker, and the mixture was stirred at a constant rate of 200 r/min in a water bath (30 °C) for 5 h. Finally, 3 mL of the silica nanoparticle solution was added, followed by stirring for 10 h to form a hydrophobic suspension, which was stirred in a fume hood to remove the formed ammonia.

We fabricated four types of ZnO NWs sensing layers to optimize the process parameters, which are named Sample 1, Sample 2, Sample 3 and Sample 4, and the process conditions are listed in Table 1. The hydrophobic engineering strategy for the construction method of the ZnO NW-based SAW is illustrated in Fig. 1b. For Sample 1, a pristine ZnO NWs sensing layer was fabricated by drop-coating a 0.2 mg/ml ZnO NWs solution, followed by drying at 45 °C for 30 min. For Sample 2, a mixture of ZnO NWs and hydrophobic silica nanoparticles was fabricated by drop-coating a mixed solution of 10 ml of ZnO NWs and 10 ml of hydrophobic silica nanoparticles and ultrasonically stirring for 2 h, followed by drying at 45 °C for 30 min. For Sample 3 (with a hydrophobic layer and a second ZnO NWs layer), 2.5 μl of hydrophobic solution was dropped onto the sensitive area and dried at 45 °C using a hot plate for 30 min to form a hydrophobic coating layer. Then, 2.5 µl of ZnO NWs aqueous solution was dropped onto the surface of the hydrophobic layer using a pipette, followed by drying at 45 °C for 30 min. Sample 4 (with a ZnO NWs layer and a second hydrophobic layer) was generated by reversing the preparation process of Sample 3 to form the ZnO NWs/hydrophobic bilayer structure.

**Table 1:** Different SAW Sensors with Different Sensing Layers

Sample	Sensing layer and construction method
Sample 1	Pristine ZnO NWs
Sample 2	Mixture of ZnO NWs and hydrophobic material
Sample 3	First step hydrophobic layer + second step ZnO NWs layer
Sample 4	First step ZnO NWs layer+ second step hydrophobic layer

Scanning electron microscopy (SEM, Sigma-300, ZEISS, Germany) with energy dispersive X-ray spectroscopy (EDS) was used to characterize the surface morphologies of the sensitive films. The water contact angles (WCAs) on the prepared sensing layer were measured at room temperature using a WCA measuring device (SDC-100, SINDIN Company). Elements and chemical bonds in the samples were analyzed using X-ray photoelectron spectroscopy (XPS, Thermo Scientific, USA).

### Fabrication of SAW sensors and sensing measurements

Two-port SAW resonators were fabricated on a 4-inch Y-X cut 128° lithium niobate (LiNbO_3_) substrate with a thickness of 500 μm. The interdigitated transducers (IDTs) were fabricated using a standard UV-light photolithography method, followed by a lift-off process with a 5 nm thick Cr layer and a 30 nm thick gold (Au) layer as the IDT electrodes. The wavelength (λ) of the SAW device was 20 μm, with 50 IDT pairs and a metallization rate of 0.5. The number of reflectors was 100. The IDT’s aperture was 200 λ, and the center distance between two opposite IDTs was 150 λ. Figure 1c shows an optical microscopy image of the fabricated two-port SAW resonators with a wavelength λ of 20 μm. An enlarged image, shown in Fig. 1c, shows the patterned IDT fingers of the fabricated SAWs.

To fabricate SAW UV sensors, the sensing material was coated onto the acoustic wave propagation area of the SAW device using a drop-coating process, followed by a drying process at 45 °C for 30 min to form the UV sensing layer.

SAW chips were packaged onto a printed circuit board and then placed inside an aluminum chamber (150 mm × 90 mm × 45 mm), as shown in Fig. 1d. The transmission characteristics (S parameters) of the SAW chip were measured using a vector network analyzer (Ceyear 3656D, China). The relative humidity (RH) levels in the box were controlled by regulating the flow ratios of dry N_2_ gas and another N_2_ source bubbled through a water bottle. The RH level was calibrated using a standard thermo hygrometer. For UV detection, a UV light-emitting diode (LED, NBet Technology Co., Ltd., China) with a wavelength λ of 365 nm was used to illuminate the sensing layer of the SAW device through a transparent observation window above the gas chamber, and the UV intensity through the observation window was measured by an LH-127 UV Light Meter. A LabVIEW program was developed to record the frequency changes as a function of time at different UV intensities and different RH levels.

## Results and discussion

**Fig. 2:**
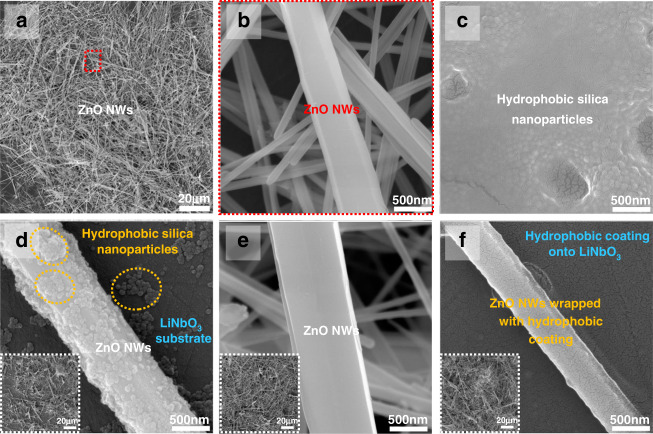
Characterization of the four types of sensitive layers on the LiNbO_3_ substrate. **a** SEM image of Sample 1 sensing layer with pristine ZnO NWs, showing the 3D network structure. **b** An image of a single ZnO nanowire from Sample 1 amplified to 500 nm. **c** The surface morphology of LiNbO_3_ after coating with a silica layer, showing that there are very small cracks and small holes on the layer surface. **d** SEM image of the Sample 2 sensing layer with mixed solution. The inset shows that the hydrophobic silica nanoparticles are unevenly distributed on the ZnO NWs and LiNbO_3_ substrate. **e** SEM image of the sensing layer of Sample 3 (with a 1 st hydrophobic layer +a 2nd ZnO NWs layer). The hydrophobic silica nanoparticles are not modified on the nanowires, the surface morphology is similar to that of the pristine ZnO NWs (Fig. 2b). **f** SEM image of the sensing layer of Sample 4 (with a 1st ZnO NW layer +a 2nd hydrophobic layer), which shows that the hydrophobic silica nanoparticles form a uniform and dense hydrophobic layer on ZnO NWs-coated LiNbO_3_

The surface morphology of the ZnO NWs coated on the surface of LiNbO_3_ was characterized using SEM, and the image is shown in Fig. 2a. Clearly, the ZnO NWs are stacked and staggered among each other to form a large number of porous interconnection networks, constructing a 3D porous structure with a large surface volume ratio, thus improving the sensing performance of the SAW sensor. Figure 2b shows an enlarged view of the ZnO NWs, revealing diameters from 50 to ~500 nm. Figure 2c shows the surface morphology of the silica nanoparticles, which were coated directly onto the LiNbO_3_ substrate, revealing a continuous and uniform layer. Figure S2 shows an optical transmission spectrum of the silica layer on a quartz substrate in a wavelength range of 200–2500 nm. It does not show an apparent difference from that of the bare quartz. Clearly, at a wavelength of 365 nm, ultraviolet light can penetrate through this silica layer.

**Fig. 3:**
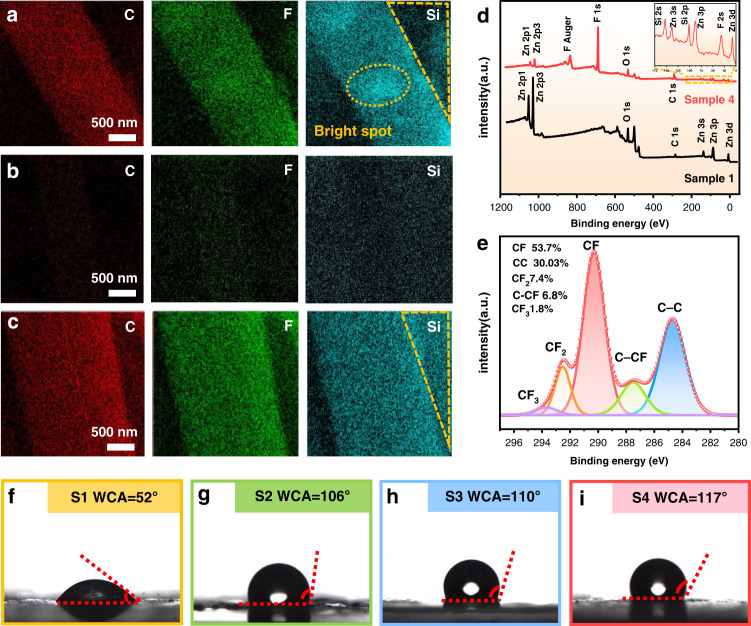
Elemental mappings of C, F, and Si elements in Sample 2, Sample 3, and Sample 4, respectively. **a** Energy spectrum of Sample 2. The light and dark spots show that the hydrophobic silica nanoparticles are unevenly distributed on the surfaces of the nanowires and LiNbO_3_ substrate. **b** The outline of the ZnO NWs in Sample 3 is not clear, which shows that the silica nanoparticles do not fully cover the ZnO NWs surface. **c** The outline of the ZnO NWs in Sample 4 is clear, which indicates that hydrophobic silica nanoparticles are evenly distributed on the surface of the ZnO NW-coated LiNbO_3_ substrate. **d** XPS survey spectra of Sample 1 and Sample 4. **e** Core level region of the C(1 s) spectrum with corresponding deconvolution peaks for Sample 4. **f**–**i** WCA images of Sample 1 (S1), Sample 2 (S2), Sample 3 (S3), and Sample 4 (S4)

Figure 2d shows the surface morphology for Sample 2 prepared with a mixed solution of ZnO NWs and a silica particle layer. The silica coating for Sample 2 is not uniform and quite loose, which was revealed from the EDS elemental mappings of C, F, and Si elements of the silica (Fig. 3a). The surface of LiNbO_3_ was not fully covered with the silica layer. Figure 2e presents the surface morphology of the sensing layer for Sample 3, which has a first layer of hydrophobic silica, followed by a second layer of ZnO NWs. In this case, a hydrophobic layer was not formed on the ZnO NWs, which was confirmed by the EDS elemental mapping of C, F, and Si, as shown in Fig. 3b. Figures 2f and 3c present the SEM image and EDS elemental mapping of Sample 4, which is first coated with ZnO NWs and then followed by a hydrophobic silica layer. The results show that hydrophobic silica nanoparticles are wrapped on the surface of ZnO NWs and thus can effectively change the surface from hydrophilic to hydrophobic.

To further characterize the hydrophobic silica nanoparticles and the hydrophobicity of the ZnO NWs, XPS analysis was used to obtain the surface chemical properties of the ZnO NWs. Figure 3d shows the XPS survey spectra of the pristine ZnO NWs (Sample 1) and the synthesized ZnO NWs wrapped with silica nanoparticles (Sample 4). Compared with Sample 1, F 1s (~685.5 eV)^35^, C 1 s (~284.8 eV), Si 2p (~101.7 eV), and Si 2 s (~153.3 eV)^36,37^ peaks were observed in Sample 4, indicating that the fluorinated and hydrophobic silica nanoparticles were successfully coated onto the ZnO NWs, which is consistent with the EDS results. Figure 3e shows the high-resolution C 1 s peak, along with its fitting results. The C 1 s peak can be deconvoluted into five regions: CF_3_, CF_2_, CF, C–CF, and C–C bonding at binding energy values of 294.0 eV, 292.7 eV, 290.3 eV, 287.3 eV and 284.7 eV, respectively, which are similar to those reported in the literature^38^. These results confirm the formation of carbon-fluorine bonds, which are hydrophobic. The ratio of F/C atoms is also an important and decisive parameter for determining the wettability of a surface, and this value was calculated (according to ref. ^38^) to be 0.739 for Sample 4, demonstrating that Sample 4 shows a good hydrophobic effect.

The hydrophilic and hydrophobic properties of the sensitive layer were further evaluated by means of static WCA measurements using the sessile drop method. Figure 3f–i show the obtained water contact angles for the four types of sensing layers. The pristine ZnO NWs (Sample 1) are hydrophilic with a WCA of ~52°. When the ZnO NWs were coated with a hydrophobic silica layer (Sample 2), the WCA increased to ~106°, showing that the sensing layer became hydrophobic. For Sample 3, with hydrophilic ZnO NWs coated onto a silica layer, the sample showed a large WCA of ~110° because the hydrophilic layer formed first on the hydrophobic LiNbO_3_ substrate. Sample 4 showed the largest WCA of ~117°, indicating that this type of 3D nanostructure (silica particles coated onto ZnO NWs) has the best hydrophobic properties.

**Fig. 4:**
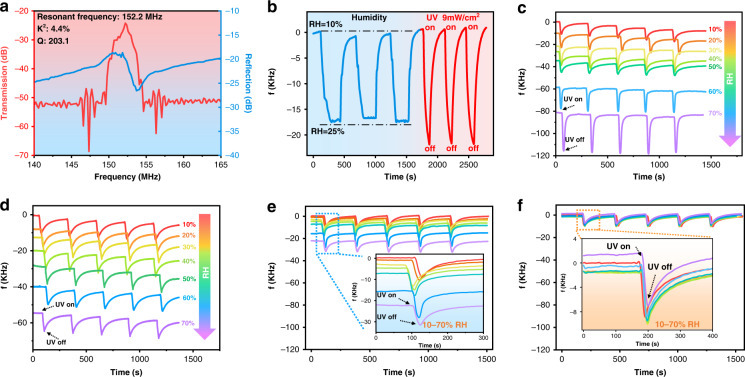
Response characteristics of SAW devices with different sensitive layers to UV and humidity. **a** Transmission (S21) and reflection (S11) spectra of the fabricated SAW device with a wavelength of 24 μm. **b** Frequency of the SAW sensor (ZnO NWs as the sensitive layer) varies with three cycles of humidity (from 10 RH% to 25 RH%) and UV (9 mW/cm^2^). **c**–**f** The frequency of the sensor (S1, S2, S3, S4) varies with humidity (from 10 RH% to 70 RH%) and UV (9 mW/cm^2^)

Figure 4a illustrates the frequency signal (S parameter) of the fabricated SAW devices with a wavelength of 24 μm. The transmission characteristics spectrum (S_21_) presents a well-defined resonant peak, indicating a resonant frequency of 152.2 MHz. The transmission amplitude of the SAW device was 27 dB. The electromechanical coupling coefficients (*k*^*2*^ values) of the devices were calculated using the following equation^39^:

1 $$k^2 = \frac{{\pi G_m\left( {f_r} \right)}}{{4NB_s\left( {f_r} \right)}}$$

where *N* is the finger pairs and *G*_*m*_*(f*_*r*_*)* and *B*_*s*_*(f*_*r*_*)* are the motional conductance and static susceptance at the resonant frequency, *f*_*r*_, respectively. The calculated *k*^*2*^ value of our SAW sensor is approximately 4.4%, which is close to previously reported values^11^, indicating that the fabricated SAW in our work has reasonably good performance.

Figure 4b shows variations in the resonant frequencies of the fabricated SAW device with only a ZnO NWs sensing layer (Sample 1) under different RH levels and UV light intensities. When the relative humidity changed from 10% to 25% for three cycles, the resonant frequencies decreased by a maximum value of ~17 KHz. The resonant frequency returned to its original value without any apparent saturation or hysteresis observed when the relative humidity decreased from 25% to 10%. These results demonstrate that ZnO NW-based SAW devices have good humidity sensing characteristics and good repeatability. This is because ZnO NWs are hydrophilic, and water molecules can be easily absorbed onto the ZnO NWs layer of the SAW device in a humid environment. This induces a significant mass loading effect on the device’s surface, which dramatically influences the transmission characteristics of the SAW device.

On the other hand, the ZnO NW-coated LiNbO_3_ SAW device (Sample 1) showed a negative shift and complete recovery with and without UV irradiation (with a UV intensity of 9 mW/cm^2^) for three cycles, demonstrating that this ZnO NW-based SAW device has good UV sensing performance with good repeatability. The UV response of the ZnO NWs SAW sensor is mainly due to the generation of free carriers (electron-hole pairs) induced by UV light, which interact with the acoustic wave field, resulting in a change in the transmission characteristics, such as the acoustic wave velocity (or resonant frequency). Without ultraviolet light, due to the inherent defects of ZnO NWs, oxygen molecules in the air will be trapped on the surfaces of the ZnO NWs, as illustrated in Fig. 1a. These captured oxygen molecules will deprive free electrons of the surface-sensitive layer and become oxygen ions, thus forming a depletion region. The relevant chemical reactions are listed in the following equations^40^.

2 $$O_2\left( g \right) + e^ - \to O_2^ - \left( {ad} \right)$$

When irradiated by ultraviolet light, electrons will be transmitted from the valence band to the conduction band of ZnO, thus forming holes in the valence band and more electron-hole pairs. Recombination of the confined electrons and holes will release the adsorbed oxygen molecules back to the atmosphere. An increase in the carrier concentration will increase the conductivity of ZnO, which will influence the acoustic wave field, resulting in changes in the transmission characteristics, such as the acoustic wave velocity and resonant frequency^40,41^.

3 $$2O^{2 - } + 2h^ + \to O_2\left( g \right)$$

Figure 4c shows the UV sensing results (at a wavelength of 365 nm and an intensity of 9 mW/cm²) for Sample 1 at different RH values from 10% to 70%. The ZnO NW-based SAW device has stable UV detection performance with good repeatability without being influenced by humidity. However, humidity will significantly affect the starting/initial resonant frequencies of the ZnO NW-based SAW devices. The larger the RH values are, the larger the frequency shifts of UV responses. The frequency shifted by ~80 KHz when the RH was 70%, demonstrating that humidity significantly influenced UV detection. The high humidity also slightly increased UV sensitivity, causing inaccurate UV detection at different levels of humidity.

When the ZnO NWs were mixed with silica particles (Sample 2), as shown in Fig. 4d, the initial resonant frequency was less significantly decreased under the same humidity conditions compared with that of the pure ZnO NWs. This is because the surfaces of ZnO NWs are coated with hydrophobic silica nanoparticles, thus forming a hydrophobic layer. However, as the surface of the ZnO NWs on the LiNbO_3_ is not uniformly coated with a whole layer of hydrophobic silica particles, it can still absorb water, thus leading to the frequency offset caused by humidity increases.

For Sample 3 (i.e., the LiNbO_3_ substrate first coated with a hydrophobic layer and then coated with a ZnO NW layer), the changes in the initial resonant frequency decreased (Fig. 4e) compared with that of the ZnO NWs coated directly onto LiNbO_3_. However, humidity still affected UV detection because the hydrophobic layer only exists below the ZnO NWs layer and can still adsorb water molecules.

Figure 4f shows the UV responses of Sample 4 (i.e., with the first step of ZnO NWs coating followed by a second silica layer) under different RH levels. When the humidity increased from 10% to 70% RH, the resonant frequencies were similar, and the frequency-UV response curves nearly overlapped, indicating that this SAW UV sensor has a unique feature to minimize humidity interference.

These results show that the 3D nanostructured sensing layer has significant effects on the hydrophobic characteristics of ZnO NW-based SAW UV sensors. The samples first coated with ZnO NWs followed by a second silica layer coating showed the best performance in minimizing the influence of humidity on the mass loading of the SAW device within the humidity range of 10–70% RH. However, it should be noted that when the humidity is greater than 70%, humidity still shows quite a large influence (Fig. S3). This is mainly because there are many microscale cracks on the surface of hydrophobic silica nanoparticles that are wrapped onto the surfaces of the ZnO NWs (Fig. 2f); thus, water molecules can infiltrate into the surface of this sensing layer through these cracks, leading to significant mass loading onto the SAW device and shifting the resonant frequency.

**Fig. 5:**
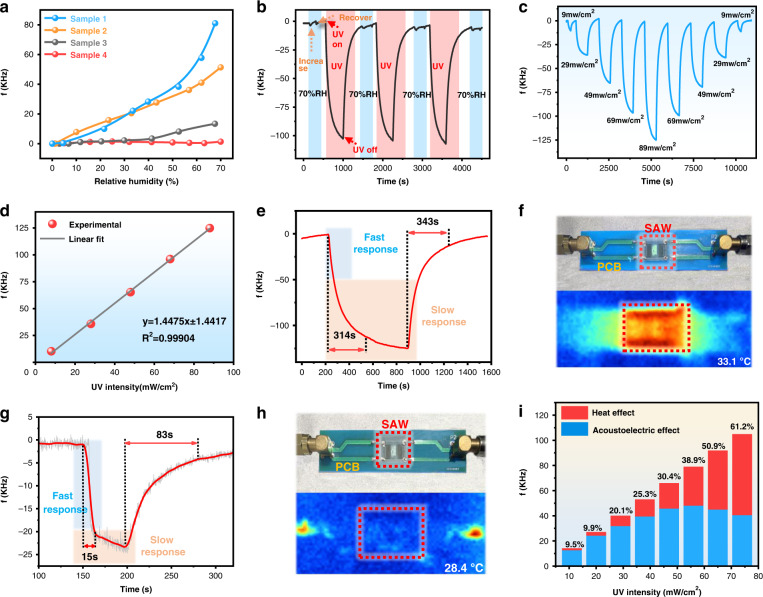
Performance test results of the proposed SAW sensor with a new sensitive layer. **a** Resonance frequency shifts as a function of relative humidity for sensors with different surface treatments. **b** Frequency shift of the humidity-insensitive UV sensor (Sample 4) after three cycles of humidity (10% RH to 70% RH) and UV treatment (89 mW/cm^2^). **c** The frequency characteristics of the Sample 4 sensor when changing the UV power from 9 mw/cm^2^ to 89 mW/cm^2^ UV over nine cycles. **d** Relative linear function of the frequency variation and UV intensity. **e** Response of the hydrophobic ZnO NWs SAW UV sensor under high UV intensity. **f** Temperature change of the ZnO NWs SAW surface under high UV intensity. **g** Response of the hydrophobic ZnO NWs SAW UV sensor under low UV intensity. **h** Temperature change of the ZnO NWs SAW surface under low UV intensity. **i** Proportion of the resonant frequency shift contribution from the SAW UV sensors due to thermal and acoustoelectric effects under different light intensities

Figure 5a summarizes the frequency responses of the SAW devices with different sensing layers under different humidity conditions. Again, SAW Sample 4 showed the best UV sensing performance without apparent humidity interferences.

Figure 5b shows the variations in the resonant frequencies of the fabricated SAW devices with ZnO NWs wrapped with a silica layer under alternating cycles of humidity and UV radiation. The results demonstrate that the SAW devices showed good UV sensing characteristics and good repeatability without apparent influence from RH.

The UV responses of the fabricated SAW device (with ZnO NWs wrapped with a hydrophobic layer) as a function of the applied UV intensity, which increased from 9 mW/cm^2^ to 89 mW/cm^2^, are shown in Fig. 5c. The results showed that the frequency change of the SAW device increased with increasing UV light, and the frequency shift was linearly correlated with the applied UV intensity. The calculated linear regression coefficient *R*^*2*^ was 0.99904, displaying excellent linearity (Fig. 5d). The maximum hysteresis of the SAW device was also calculated, and the value was less than 1.6%. The UV light sensitivity of the SAW devices was defined as^42^:

4 $$S_{UV} = \frac{1}{{f_r}}\frac{{\Delta f}}{{\Delta I_{UV}}}$$

where Δ*I*_*UV*_ is the UV intensity. The sensitivity of the hydrophobic ZnO NW-based SAW device was 9.53 ppm (mW/cm^2^)^−1^.

The response time and recovery time are defined as the time for 90% signal change in the full-scale response during UV source switching on and off. As shown in Fig. 5e, the SAW sensor with ZnO NWs wrapped with a hydrophobic layer showed response/recovery times of 314 s and 343 s, which are larger than those of the pristine ZnO NW-based SAW device (164 s and 262 s as shown in Fig. S4).

To investigate the physical mechanisms of such high response/recovery times of the ZnO NW-based SAW devices on LiNbO_3_, we measured the temperature effects at various UV intensities using an infrared thermometer with an ambient temperature of ~28.3 °C. Figure 5f shows that the temperature increased from ~28.4 °C to 33.1 °C under high UV power (89 mW/cm^2^) for an increase of ~4.7 °C. The theoretical frequency shift of the SAW device caused by temperature changes can be written using the following formula^43^:

5 $$\Delta f_T = f_0 \times TCF \times \Delta T$$

where *f*_0_ is the center frequency of the SAW UV sensor, *TCF* is the frequency temperature coefficient of the substrate material, Δ*T* is the change in temperature, Δ*f* is the frequency offset of the device, and Δ*f*_*T*_ is the frequency offset caused by temperature. The frequency shift of the SAW device caused by the temperature effect is approximately 64.2 KHz. This value is ~61.2% that of the total frequency change, indicating that the thermal effect is significant during UV detection at high power. As the temperature increased, the decrease in resonant frequency was very slow; thus, the response time was relatively long. To verify this, we also conducted UV detection using a low power of 11 mW/cm^2^. The corresponding response time was only 15 s (Fig. 5g), which was much shorter than that of the SAW at a high power. Figure 5h shows that the temperature is almost unchanged under this low UV intensity, with an increase of only 0.1 °C. At a lower UV intensity, the frequency shift caused by the temperature is 1.3 KHz, which is only 9.5% of the total frequency shift. Moreover, we measured the proportion of the contribution to the resonant frequency shift of the SAW UV sensors due to thermal and acoustoelectric effects under different light intensities, and the obtained results are shown in Fig. 5i. From these results, we can conclude that the acoustoelectric and heat effects caused frequency shifts in the ZnO NW-based SAW device on the LiNbO_3_ substrate. The UV response characteristics of the SAW device are divided into two processes: the fast UV response process caused by the change in conductivity and the slow temperature response process caused by the temperature effect due to the TCF. The frequency shift caused by conductivity changes (i.e., the acoustoelectric effect) is dominant at low UV light intensity, while the thermal effect will be enhanced when using a high-power UV light source. It should be noted that the TCF (calculated by the formula $TCF = \frac{1}{{f_0}}\frac{{df}}{{dT}}$)^44^ of our SAW device without a sensitive layer is −84.87 ppm/°C (Fig. S5a), which is similar to the TCF of LiNbO_3_ reported in a previous study^45^. The TCF of the device with a sensitive layer (e.g., ZnO NWs wrapped with hydrophobic silica nanoparticles, Sample 4) is −87.83 ppm/°C (Fig. S5b), showing that the TCF of the sensor will not be changed by coating with a sensitive layer.

As discussed above, the SAW UV sensing responses can be divided into a fast response process caused by the acoustoelectric effect and a slow response process caused by the thermal effect. We can further improve the UV response speed of such devices by improving the response speed of the acoustoelectric effect (as the thermal effect will not increase this reading too much). Graphene quantum dots (GQDs) were previously introduced into ZnO NWs, which can provide more oxygen adsorption sites for the ZnO NWs^10^. This new composite (i.e., ZnO NWs & GQD layer + hydrophobic layer) will accelerates processes of adsorption and desorption of oxygen molecules on the surface of the device, which shortens the response time and recovery time, as shown in Fig. S6. Using this new composite sensitive layer, under high light intensity (89 mW/cm^2^), the response time was reduced to 134 s (Fig. S6a) and the recovery speed was reduced to 189 s. Notably, under a low light intensity (11 mW/cm^2^), the response speed was reduced to 9 s (Fig. S6b) and the recovery speed was reduced to 40 s. Table SI summarizes the performance of literature-reported SAW-based UV detectors and also the sensor in this work, revealing that our humidity-insensitive SAW UV sensor has relatively better sensitivity and a faster response time under a low light intensity compared with those of many previous studies^22,46–52^.

**Fig. 6:**
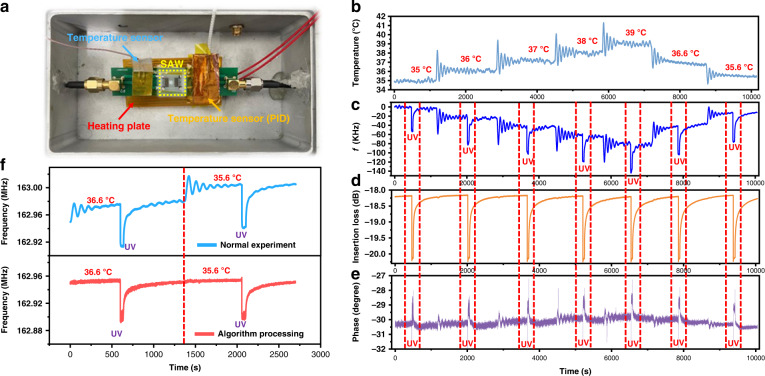
Test system with temperature interference, and response characteristics of SAW devices to temperature and ultraviolet. **a** Photo of the SAW UV test device with temperature interference. **b** Variations in the interference temperature applied to the SAW UV sensor over time. **c**–**e** Changes in SAW frequency, insertion loss and phase under interference of temperature and UV treatment. **f** Relationship between frequency and time for the SAW UV sensor under temperature interference before and after algorithm processing

Since the thermal effect will change the device’s resonant frequency, the effect of environmental temperature on the device should be considered to make accurate measurements of UV intensity. In this study, we proposed using an artificial intelligence (AI) algorithm (i.e., a random forest algorithm) to distinguish/differentiate the environmental temperature effect and UV effects. Figure 6a shows the environmental temperature control system and the UV testing system, which are mainly composed of a proposed SAW sensor, a heating plate to change the environmental temperature, a thermocouple temperature sensor to detect the temperature, a PID temperature controller, and a UV light source. We applied different temperatures by heating (35, 36, 37, 38, and 39 °C) the SAW UV sensor with or without UV radiation. Figure 6b shows the temperature changes over time detected using the thermocouple temperature sensor, and these have been used as the applied interference temperatures to the SAW UV sensor. It is clear that the interference temperatures increased from 35 to 39 °C and then decreased to 35.6 °C from 0 s to 10000 s.

Figure 6c shows the frequency shifts of the SAW device with the interference temperatures and UV applied simultaneously. The results show that both temperature and UV light can shift the resonant frequency. However, the insertion loss and phase angles of the SAW device were not changed much by the environmental temperature, as shown in Fig. 6d, e, whereas UV light can significantly change the insertion loss and phase of the SAW device.

Therefore, we used the frequency, insertion loss, phase and amplitude values as the key features, and we proposed a random forest algorithm to distinguish/differentiate the environmental temperature effect and UV effect. We used 31274 experimental data points as the training set and 11916 data points as the test set, and we trained the model with frequency, amplitude, phase, and insertion loss data as the key features. Using this method, we effectively distinguished/differentiated the temperature effects from the UV effects and minimized the influences of environmental temperature. Figure 6f shows that using our proposed AI algorithm for the SAW device, we can minimize the frequency change caused by temperature and truly show the UV-influenced results.

## Conclusion

In this work, we proposed a novel strategy by regulating the properties of ZnO NWs from a hydrophilic state to a hydrophobic state using a new mixed material design with hydrophobic silica nanoparticles wrapped on the surface of the ZnO NWs. This newly designed sensing layer endows the ZnO NW-based UV sensor with significantly reduced humidity interference in the humidity range of 10–70% RH, showing a UV sensitivity of 9.53 ppm (mW/cm^2^)^−1^, with high linearity (*R*^*2*^ value is 0.99904), small hysteresis (less than 1.65%) and good repeatability. The construction method of using mixed materials to minimize the humidity effect was investigated, and the sensing mechanisms of the acoustoelectric effect and heat effect for such SAW UV detection were also investigated. Moreover, we proposed an AI algorithm (a random forest algorithm) to distinguish/differentiate the environmental temperature and UV effects. This work solves a long-term dilemma for the severe humidity effect on ZnO NW-based sensors and has great potential to monitor UV levels under various environmental humidity conditions.

## Supplementary information

Supplementary information
