# Supplementary material for: A new strategy to minimize humidity influences on acoustic wave ultraviolet sensors using ZnO nanowires wrapped with hydrophobic silica nanoparticles
Source: Microsyst Nanoeng. 2022 Nov 15;8:121. doi: 10.1038/s41378-022-00455-2 (PMC9666537; doi:10.1038/s41378-022-00455-2)
Supplement: Supplementary file 1 — Supplementary information [file 41378_2022_455_MOESM1_ESM.docx]

**A New Strategy to Minimize Humidity Influences on Acoustic Wave Ultraviolet Sensors Using ZnO Nanowires Wrapped with Hydrophobic Silica Nanoparticles**

*Yihao Guo^1^, Jian Zhou^*,1^, Zhangbin Ji^1^, Yanghui Liu^1^, Rongtao Cao^1^, Fengling Zhuo^1^, Kaitao Tan^1^, Huigao Duan^1^, Yongqing Fu^2^*

1. College of Mechanical and Vehicle Engineering, Hunan University, Changsha 410082, China
2. Faculty of Engineering and Environment, Northumbria University, Newcastle upon Tyne, NE1 8ST, United Kingdom

**Corresponding author E-mail:** [jianzhou@hnu.edu.cn](mailto:duanhg@hnu.edu.cn)

1. **Synthesis processes of composite materials based on ZnO NWs wrapped with hydrophobic silica nanoparticles**


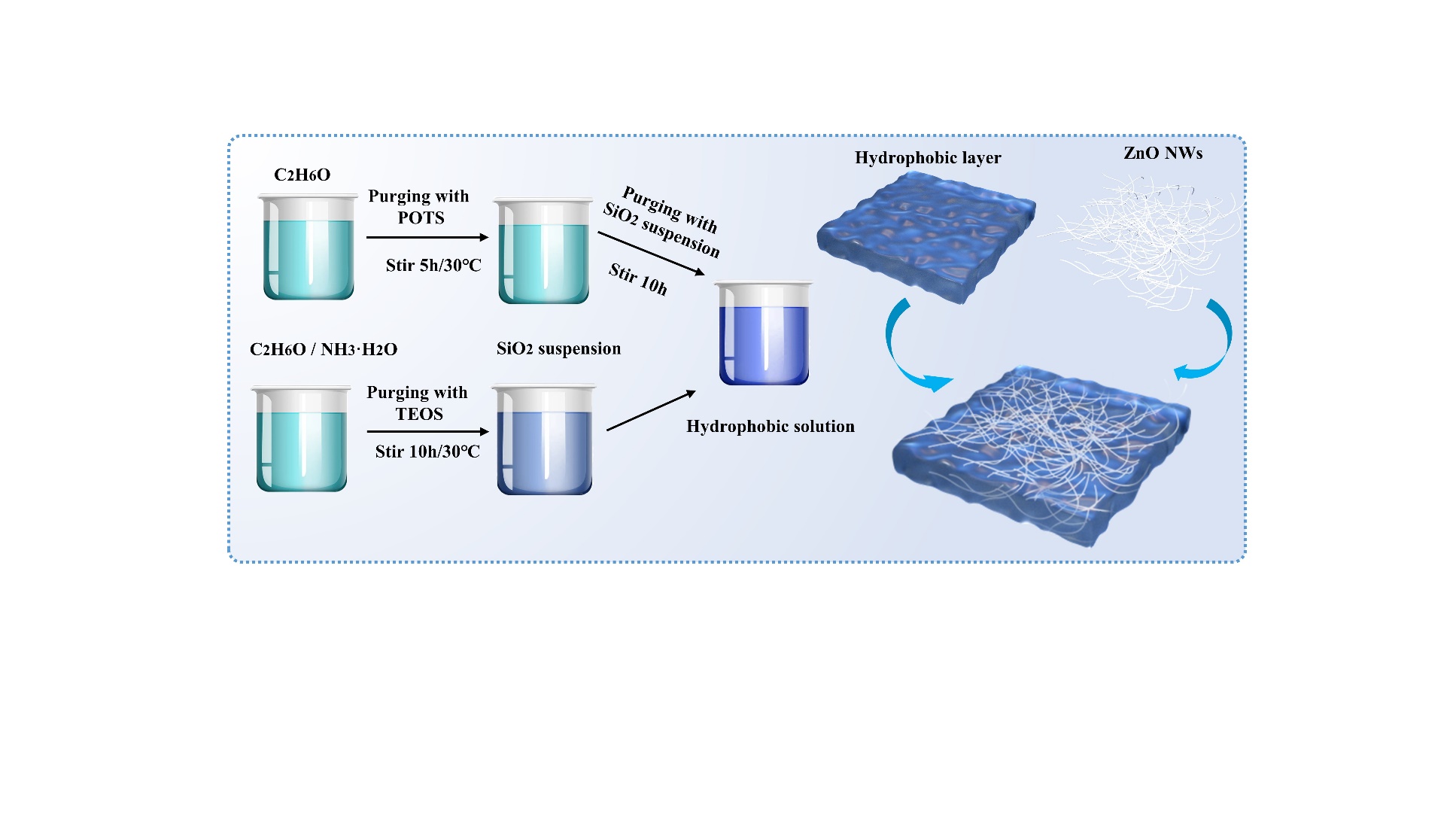


Figure S1. Illustrations of synthesis processes for sensitive layers of composite materials based on ZnO NWs wrapped with hydrophobic silica nanoparticles

1. **Optical transmission spectrum of hydrophobic coating on quartz.**


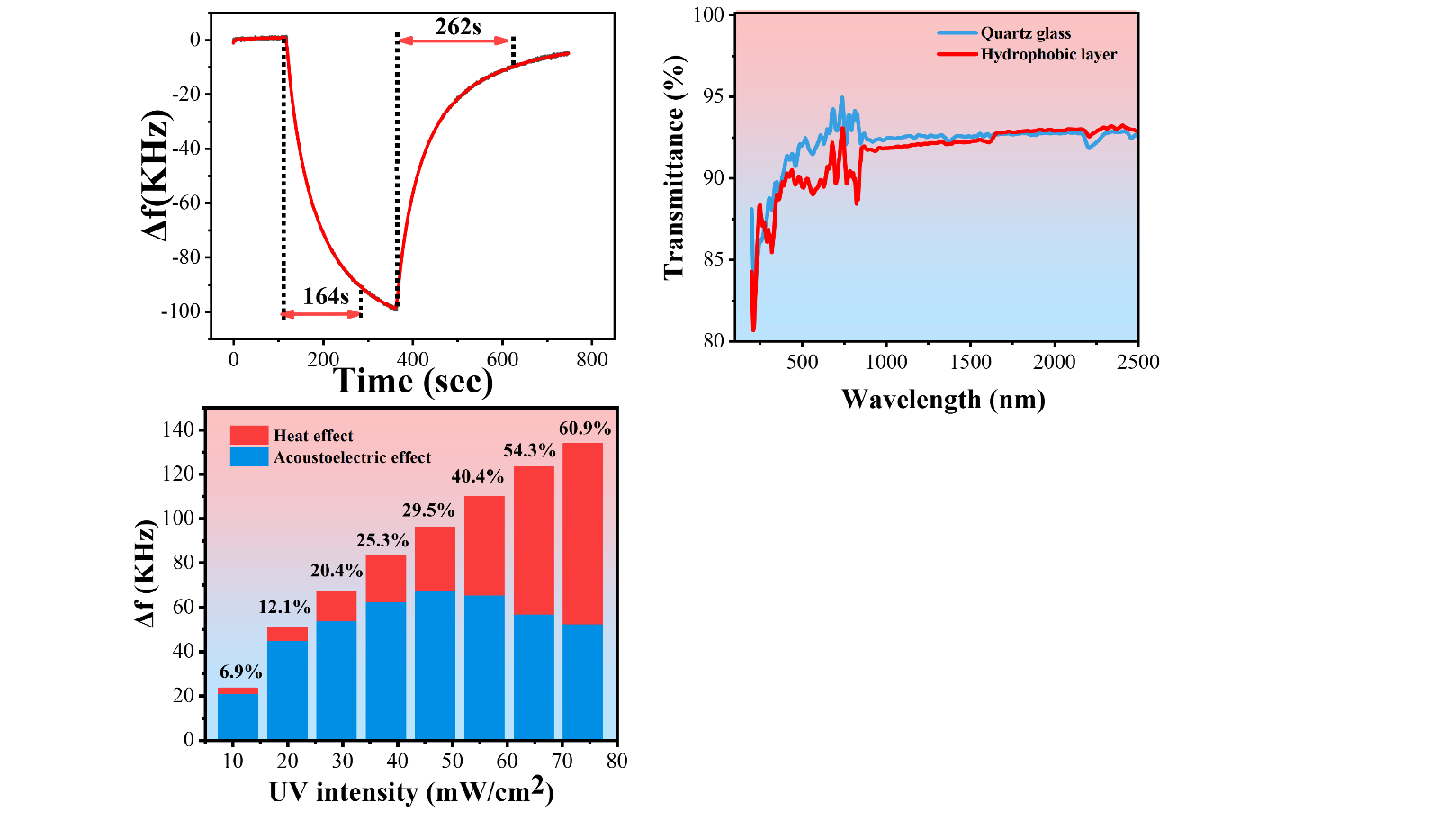


Figure S2. Optical transmission spectra of quartz and hydrophobic silica layer on the quartz

1. **Frequency shifts of sample 4 for UV sensing, with the relative humidity values of 80% RH and 87% RH**


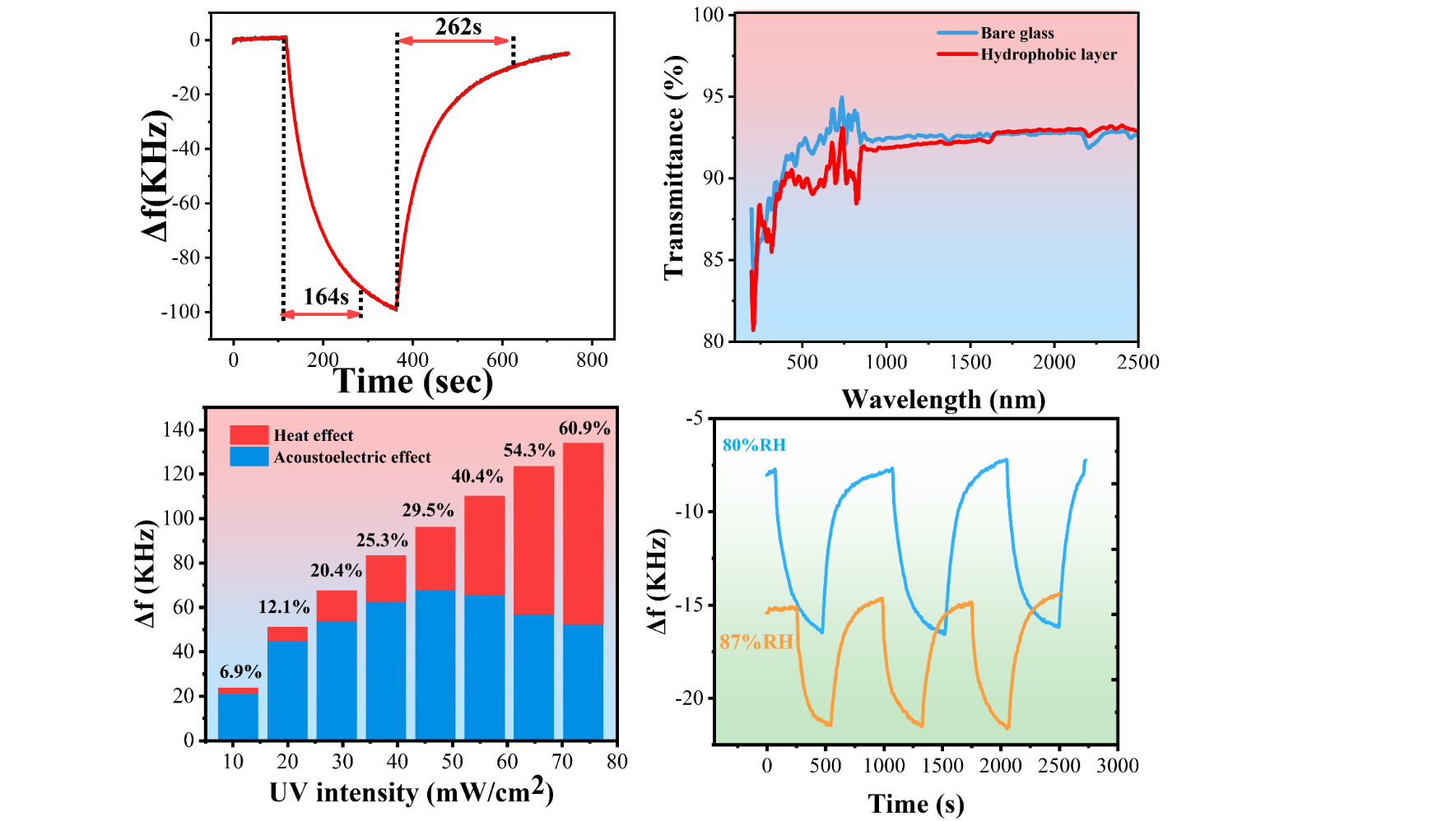


Figure S3 Frequency shift of sample 4 for UV sensing with relative humidity values of 80% RH and 87% RH

1. **Response/recovery times of SAW UV sensor with ZnO nanowires as a sensitive layer.**

**
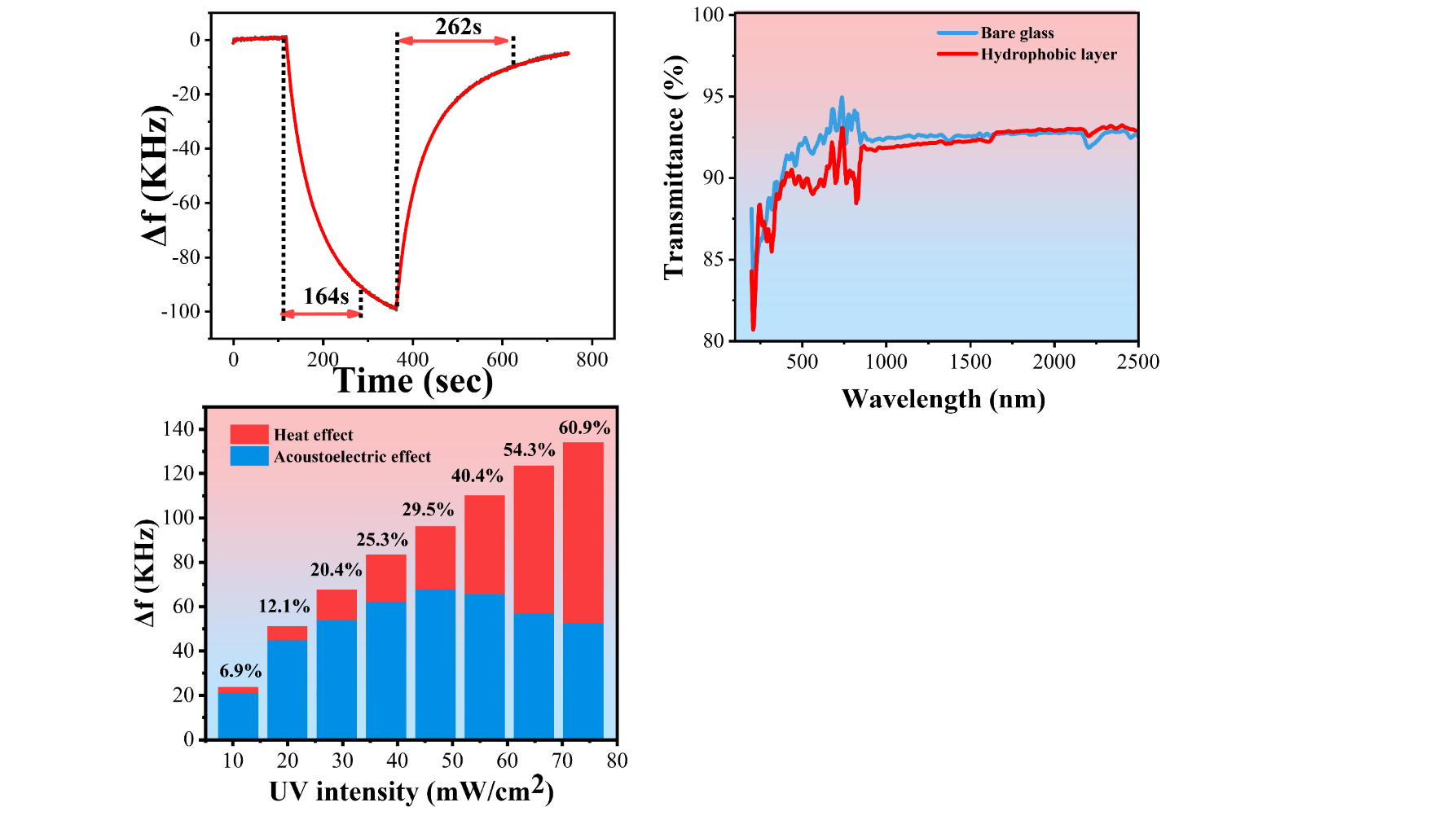
**

Figure S4. Response/recovery times of UV detector with ZnO NWs layer at a UV intensity of 89 mW/cm^2^

1. **TCF testing results of SAW devices before and after deposited with the sensitive layer.**

**
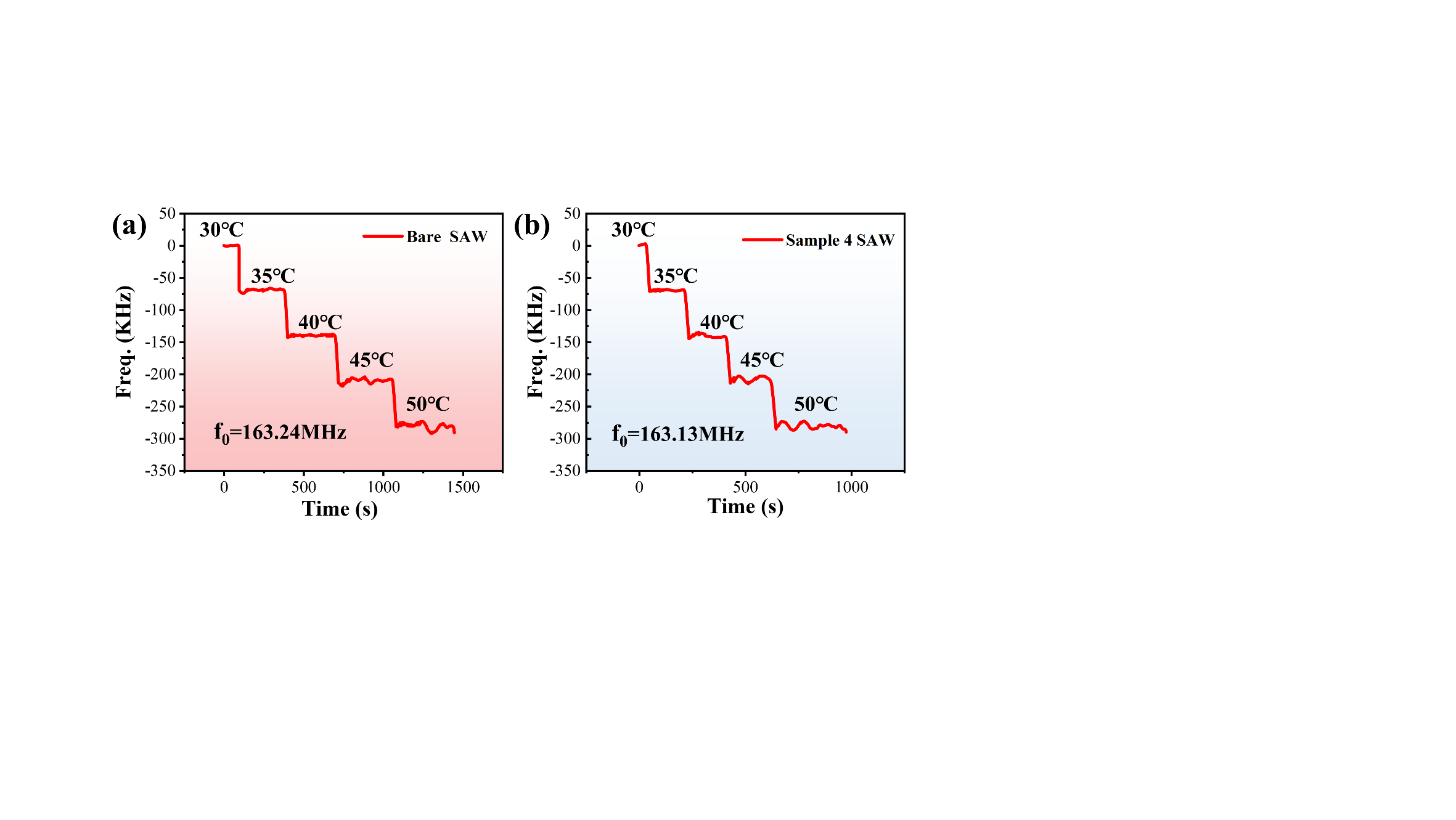
**

Figure S5. (a) Frequency shifts of SAW device without a sensitive layer as a function of temperature; (b) Frequency shifts of SAW device with a sensitive layer of ZnO NWs wrapped with hydrophobic silica nanoparticles as a function of temperature.

1. **Frequency response of ZnO NWs & GQDs +hydrophobic layer-based SAW UV sensor at a high/low UV intensities.**

**
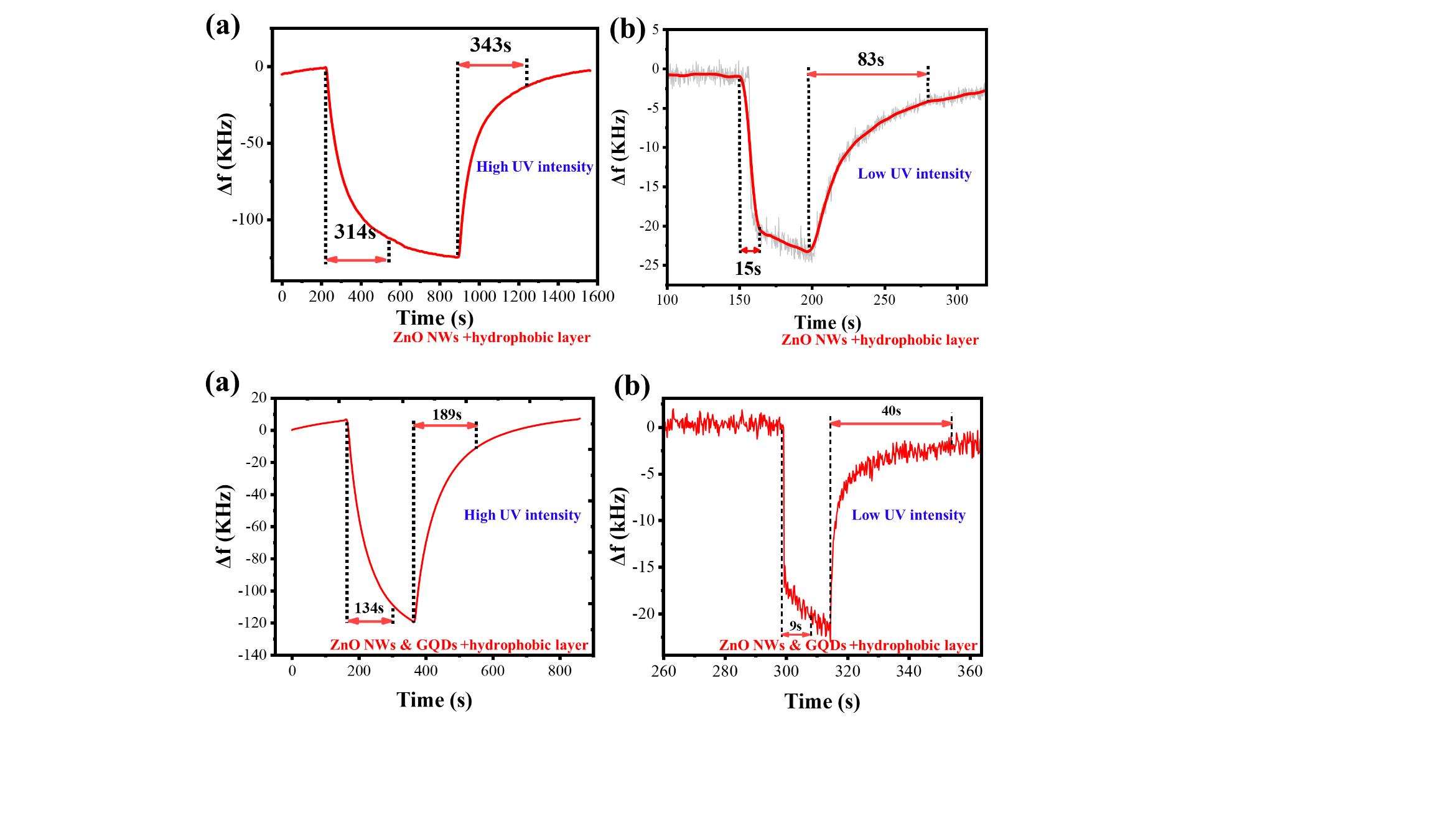
**

Figure S6. (a) Frequency responses of hydrophobic ZnO NWs & GQDs + hydrophobic based SAW UV sensor at a high UV intensity; (b) Frequency responses of hydrophobic ZnO NWs & GQDs + hydrophobic based SAW UV sensor at a low UV intensity.

1. **Performance of reported SAW-based UV detectors.**

Table SI Performance of reported SAW-based UV detectors in literature and in this study.

| **Sensitive material** | **Piezoelectric**  **material** | **Wavelength**  **(nm)** | **Resonant**  **mode** | **Sensitive**  **(mW/cm^2^)^-1^** | **Respond**  **time (s)** | **Ref** |
| --- | --- | --- | --- | --- | --- | --- |
| ZnO nanorod | ZnO | 365 | Sezawa | 0.15 | 3 (low intensity) | 1 |
| ZnO Nanorod | ST-cut quartz | 365 | Rayleigh | 0.16 | 40 (low intensity) | 2 |
| ZnO film | ZnO | 365 | Rayleigh | 0.04 | 10 (low intensity) | 3 |
| ZnO film | ZnO | 365 | Rayleigh | 0.04 | 2.4 (low intensity) | 4 |
| ZnO film | ST-cut quartz | 325 | - | 0.03 | 2 (low intensity) | 5 |
| ZnO nanorod | 128°*YX*-cut LiNbO_3_ | 365 | Rayleigh | 0.08 | 10 (low intensity) | 6 |
| ZnO nanowire | 128°*YX*-cut LiNbO_3_ | 365 | Rayleigh | 8.52 | - | 7 |
| ZnO nanomaterial | 128°*YX*-cut LiNbO_3_ | 365 | Rayleigh | 9.58 | 10 (low intensity) | 8 |
| This work | 128°*YX*-cut LiNbO_3_ | 365 | Rayleigh | 9.53 | 9 (low intensity)  134(high intensity) | **This work** |

**Reference:**

1 Guo, Y. et al*.* Ultraviolet sensing based on nanostructured ZnO/Si surface acoustic wave devices. *Smart Mater. Struct.* **24**, 125015 (2015).

2 Li, W. et al. Highly sensitive ultraviolet sensor based on ZnO nanorod film deposited on ST-cut quartz surface acoustic wave devices. *Surf. Coat. Technol.* **363**, 419-425 (2019).

3 Wang, W.-B. et al. Transparent ZnO/glass surface acoustic wave based high performance ultraviolet light sensors. *Chin. Phys. B* **24**, 057701 (2015).

4 Wang, W. et al*.* Thermal annealing effect on ZnO surface acoustic wave-based ultraviolet light sensors on glass substrates. *Appl. Phys. Lett.* **104**, 212107, (2014).

5 Tsai, W.-C. et al*.* Room temperature fabrication of ZnO/ST-cut quartz SAW UV photodetector with small temperature coefficient. *Opt. Express.* **23**, 2187-2195 (2015).

6 Wang, W.-S. et al. A ZnO nanorod-based SAW oscillator system for ultraviolet detection. *Nanotechnology* **20**, 135503 (2009).

7 Peng, W. et al. Surface acoustic wave ultraviolet detector based on zinc oxide nanowire sensing layer. *Sens. Actuator A Phys.* **184**, 34-40 (2012).

8 Peng, W. et al. Study on the performance of ZnO nanomaterial-based surface acoustic wave ultraviolet detectors. *J. Micromech. Microeng.* **23**, 125008 (2013).
